# Development of a Single-Step Antibody–Drug Conjugate Purification Process with Membrane Chromatography

**DOI:** 10.3390/jcm10030552

**Published:** 2021-02-02

**Authors:** Juan Carlos Cordova, Sheng Sun, Jeffrey Bos, Srinath Thirumalairajan, Sanjeevani Ghone, Miyako Hirai, Ricarda A. Busse, Julia S. v. der Hardt, Ian Schwartz, Jieyu Zhou

**Affiliations:** 1 Abzena, 360 George Patterson Boulevard, Bristol, PA 19007, USA; JuanCarlos.Cordova@abzena.com (J.C.C.); apollosunsheng@gmail.com (S.S.); bosjeffrey@gmail.com (J.B.); sri@seagen.com (S.T.); sanghone20@gmail.com (S.G.); 2 Seagen, 21717 30th Drive S.E., Bothell, WA 98021, USA; 3 Sartorius Stedim Biotech GmbH, August-Spindler-Straße 11, 37079 Göttingen, Germany; Miyako.Hirai@sartorius.com (M.H.); Ricarda.Busse@sartorius.com (R.A.B.); Julia.vonderHardt@sartorius.com (J.S.v.d.H.); 4 Sartorius North America Inc., 565 Johnson Avenue, Bohemia, NY 11716, USA; Ian.Schwartz@sartorius.com

**Keywords:** antibody–drug conjugate, membrane purification, pyrrolobenzodiazepine (PBD) dimer, cation exchange (CEX), hydrophobic interaction (HIC), tandem-mode purification

## Abstract

Membrane chromatography is routinely used to remove host cell proteins, viral particles, and aggregates during antibody downstream processing. The application of membrane chromatography to the field of antibody-drug conjugates (ADCs) has been applied in a limited capacity and in only specialized scenarios. Here, we utilized the characteristics of the membrane adsorbers, Sartobind^®^ S and Phenyl, for aggregate and payload clearance while polishing the ADC in a single chromatographic run. The Sartobind^®^ S membrane was used in the removal of excess payload, while the Sartobind^®^ Phenyl was used to polish the ADC by clearance of unwanted drug-to-antibody ratio (DAR) species and aggregates. The Sartobind^®^ S membrane reproducibly achieved log-fold clearance of free payload with a 10 membrane-volume wash. Application of the Sartobind^®^ Phenyl decreased aggregates and higher DAR species while increasing DAR homogeneity. The Sartobind^®^ S and Phenyl membranes were placed in tandem to simplify the process in a single chromatographic run. With the optimized binding, washing, and elution conditions, the tandem membrane approach was performed in a shorter timescale with minimum solvent consumption and high yield. The application of the tandem membrane chromatography system presents a novel and efficient purification scheme that can be realized during ADC manufacturing.

## 1. Introduction

Antibody–drug conjugates (ADCs) have gained great attention in oncology research and clinical studies as a novel strategy for targeted drug delivery. ADC development began in the early 1960s [1,2]. Knowledge accumulated over the past two decades, including a number of discontinued clinical studies, has resulted in 10 ADCs being commercially approved by the U.S. Food and Drug Administration (FDA), including: brentuximab vedotin (Adcetris™), ado-trastuzumab emtansine (Kadcyla™), inotuzumab ozogamicin (Besponsa™), gemtuzumab ozogamicin (Mylotarg™), Moxetumomab pasudotox (Lumoxiti™), polatuzumab vedotin-piiq (Polivy™), Enfortumab vedotin (Padcev™), Trastuzumab deruxtecan (Enhertu™), Sacituzumab govitecan (Trodelvy™), and belantamab mafodotin-blmf (Blenrep™).

ADCs are a highly complex class of molecules that consist of three parts: antibody, linker, and small-molecule drug. In contrast to antibody production, the conjugation of antibodies with small-molecule linker-drugs and subsequent purification steps are unique to ADC production. For ADC process development and manufacturing, the drug-to-antibody ratio (DAR), DAR species composition, free linker-drug, aggregates, and fragments are critical quality attributes that need to be carefully examined and controlled.

Currently, ultrafiltration/diafiltration (UF/DF) has been widely used in the ADC process to remove reaction-related impurities including solvents and impurities related to the free linker-drug [3,4]. Although the molecular weight of the free linker-drug is significantly lower than the molecular weight cut-off (MWCO) of the UF/DF membrane, due to the drug self-association or nonspecific interactions among the free linker-drug, ADC, and UF/DF membrane, very potent cytotoxic drugs such as pyrrolobenzodiazepine (PBD) dimers might not be cleared sufficiently by UF/DF. In cases where UF/DF is insufficient to remove all of the free linker-drug impurities, additional purification steps such as activated carbon filtration or chromatography are usually required [5,6]. These additional purification steps can complicate the ADC manufacturing and compromise the overall product yield, not to mention the potential scalability and leachability issues associated with carbon filtration.

In addition to free-drug removal, if aggregates or undesired DAR variants are generated during the conjugation process, chromatographic purification steps are often required. Resin-based conventional chromatography such as hydrophobic interaction (HIC), cation exchange (CEX), and mixed-mode ceramic hydroxyapatite (CHT) have been used in aggregate removal or DAR polishing [7,8,9]. However, developing a preparative resin-based chromatography method can be challenging. In particular, the flow rate and diffusion limitations associated with packed-bed chromatography can increase the risk of protein denaturation due to long contact time on the resin surface and the harsh conditions used to elute protein, both of which can cause significant product losses. Overall, the complexity of conventional chromatography options, as well as the high cost of the resin, column-packing, cleaning, and validation requirements, have driven the search for more flexible, cost-effective, and convenient alternatives.

Membrane chromatography can have a higher capture efficiency and higher productivity than conventional resin-based chromatography, and shows the most promising applications for the recovery, isolation, and purification of biomolecules [10,11,12]. Due to the predominance of convective solute mass transport within the adsorptive membrane, membrane chromatography has the potential to operate at much higher flow rates than packed columns, which could reduce the degradation and denaturation of biomolecules, as well as the buffer volume required for a given purification step. Despite such advantages, membrane chromatography is only routinely used to remove host cell proteins, viruses, endotoxins, and DNA in the flow-through mode in downstream processes [12]. The application of membrane chromatography to the field of ADCs has been applied in a limited capacity and in only specialized scenarios [12].

Engineering cysteines at specific sites in antibodies offers an attractive solution to generate well-defined ADCs, while minimizing their systemic toxicity [13]. This site-specific conjugation technology has been applied for the generation of many different ADCs and immunoconjugates for clinical studies. Here, the development of CEX and HIC membrane chromatography for a site-specific ADC process is described using an engineered cysteine-mAb with PBD dimer as a model conjugation system. We utilized the characteristics of the membrane adsorbers, Sartobind^®^ S and Phenyl (Sartosious Stedim Ltd., Göttingen, Germany), for free payload and aggregate clearance while polishing the ADC in a single chromatographic run. The Sartobind^®^ S membrane was used to remove excess free payload, while the Sartobind^®^ Phenyl was used to polish the ADC by clearance of undesired DAR species and aggregates. In addition, the Sartobind^®^ S and Phenyl membranes were placed in tandem to integrate the overall ADC purification processes in a single chromatographic run. With the optimized binding, washing, and elution conditions, the tandem membrane approach was performed in a shorter timescale with minimum solvent consumption with high yield. Key process parameters such as product yield, free linker-PBD and aggregate removal efficiency were evaluated.

## 2. Materials and Methods

### 2.1. ADC Preparation

In this study, the cysteine-engineered human monoclonal antibody (IgG1, pI 8.45, 150 kDa) used for conjugation experiments, was manufactured by Abzena (San Diego, CA, USA). The maleimidocaproyl valine citrulline p-aminobenzyloxycarbonyl monomethyl auristatin E (MC-vc-PAB-MMAE) [14] or maleimido-pyrrolobenzodiazepine (PBD) dimer [15] were prepared by Abzena (Bristol, PA, USA) using the methods previously described. The ADC was produced following a previously developed method with modifications [13]. Briefly, the conjugates were manufactured by first reducing the cys-mAb with 50 equivalents of dithiothreitol (DTT) at room temperature overnight. The cys-mAb was then diafiltered into a conjugation reaction buffer (50 mM sodium phosphate, pH 7.0, 1 mM EDTA) using a 30 kDa molecular weight cut-off (MWCO) Hydrosart^®^ ultrafiltration membrane (Sartosious Stedim Ltd., Göttingen, Germany). The interchain disulfides were reformed by adding 12 equivalents of dehydroascorbic acid (DHAA) for 3 h at room temperature. The uncapped cys-mAb was then conjugated to 3.5 equivalents of linker-payloads (linker-PBD or linker-MMAE) with 20% (*v/v*) propylene glycol added as cosolvent.

### 2.2. Dynamic Binding Capacity Study

To evaluate the membrane binding capacity, the cys-mAb, MMAE, or PBD conjugates in reaction buffer (50 mM sodium phosphate, pH 7.0) were diluted with 20 mM MES (2-(N-morpholino)ethanesulfonic acid) pH 6.0 or 25 mM sodium phosphate with 1 M ammonium sulfate pH 7.0 and loaded onto the Sartobind^®^ nano, 3 mL, 8 mm bed height S or Phenyl membranes (Sartorius), respectively.

The binding capacity studies were carried out as follows: The membranes were first flushed with equilibration buffer for 10 membrane volumes (MVs) at a residence time of 1 min. The load material was then applied at the same residence time until 10% breakthrough was observed by monitoring the UV absorbance at 280nm. The membranes were then washed with 10 MVs of equilibration buffer, and the proteins were stripped with 5 MVs of elution buffer. After stripping and regeneration, the membrane was sanitized with 10 MVs of sodium hydroxide at a residence time of 1 min, neutralized with 20 mM sodium phosphate buffer pH 7.0, and stored in 20% *v/v* ethanol.

The amount of sample loaded to the membrane was calculated according to the following equation: where C_0_ is the protein concentration in the sample (mg/mL), V_L_ is accumulated volume per fraction (mL), V_0_ is system void volume (mL), and V_c_ is the membrane volume (mL).

Sample binding capacity = (C_0_ (V_L_ − V_0_))/V_c_.

### 2.3. Purification Development

For small-scale purification development, an ÄKTA Explorer was used for screening runs. The conjugates were purified using Sartobind^®^ S and/or Phenyl membrane devices, both 3 mL, 8 mm, in bind and elute mode. ÄKTA Pilot system was used to assess scalability where conjugates were purified using Sartobind^®^ S 75 mL membrane and Sartobind^®^ Phenyl 150 mL in bind and elute mode. For all experiments, flow rates were 1 membrane volumes per minute (MV/min).

### 2.4. Sample Preparation for Free Payload Species Quantification with LC-MS/MS

The clearance of free linker-payload from Sartobind^®^ S membrane was investigated utilizing an LC-MS/MS approach. The Sartobind^®^ S membrane was first loaded with 16 μg/mL of linker-PBD, then washed up to 15 MVs with either 20 mM MES buffer (pH 6.0) or the MES buffer with 10% propylene glycol. Finally, the membrane was washed with 3 MVs of 20 mM MES, 350 mM sodium chloride buffer, pH 6.0. Each MVs wash was collected separately for LC-MS/MS analysis.

An acetonitrile precipitation method was used before the LC-MS/MS analysis to extract the free payload species and remove salts or protein species. Specifically, 100 μL of sample was mixed 1:9 with acetonitrile (ACN) prior to the centrifugation at 15,000× *g* for 20 min. Then, the supernatant was transferred into a new 1.5 mL Eppendorf tube. The solvent was completely removed by SpeedVac. The dried sample was dissolved in 20 µL of H2O/ACN (50:50 *v/v*) and analyzed via LC-MS/MS using an Agilent 1200 HPLC interface to SCIEX Triple Quad 6500 MS system. Peak separation was achieved using a Phenomenex Gemini C18 column, 3 µm, 110 Å, 4.6 × 50 mm with mobile phase A (0.1% formic acid in water) and B (0.1% formic acid in acetonitrile) with a gradient of 0–0.5 min 30% B, 0.5–3.0 min 30–100% B, 3.0–3.2 min 100% B, 3.2–3.25 min 100–30% B, and 3.25–4.0 min 30% B at a flow rate of 1.0 mL/min (column temperature of 25 °C). The retention times of linker-PBD was 2.15 min. The multiple reaction monitor (MRM) transition in MS was 1569.5/1507.6 Da for linker-PBD. Compound-dependent MS parameters were 31 for decluttering potential (DP), 10 for entrance potential (EP), 33 for collision energy (CE), and 8 for collision cell exit potential (CXP). The MS instrument dependent parameters were 9 L/h for collision gas (CAD), 35 L/h for curtain gas (CUR), 50 L/h for nebulizer gas (GS1), 0 L/h for turbo gas (GS2), 5.5 kV for ion-spray voltage (IS), and 600 °C for ion-spray temperature (TEM). The standard curve samples for quantitation was 5–250 ng/mL for linker-PBD. The limit of detection (LOD) and limit of quantification (LOQ) of linker-PBD were determined to be 11.01 and 36.70 ng/mL, respectively.

### 2.5. Characterization of ADCs: SEC and HIC Method

To determine aggregation, conjugates were analyzed by size-exclusion chromatography (SEC) (column: TSKgel G3000SWxl 7.8 mm × 30 cm, 5 μm (Tosoh Bioscience; P/N: 08541)) using 0.2 M sodium phosphate 0.2 M potassium chloride, pH 6.5 with 15% (*v/v*) isopropyl alcohol as mobile phase. An injection volume of 10 μL was loaded to the column at a constant flow rate of 0.35 mL/min. Chromatographs were integrated based on elution time to calculate the purity of monomeric conjugate species.

The drug-to-antibody ratio (DAR) was evaluated by HIC on a high-performance liquid chromatography (HPLC) system (Agilent 1260 HPLC system, TSKgel Butyl-NPR column 4.6 mm × 3.5 cm, 2.5 μm (Tosoh Bioscience; P/N: 14947)). The HIC method used 1.5 M ammonium sulfate in 25 mM potassium phosphate pH 7.0 (mobile phase A) and 25 mM potassium phosphate pH 7.0 containing 25% isopropanol *v/v* (mobile phase B) run at a flow rate of 0.8 mL/min over a 12-min linear gradient with UV monitoring at 254 and 280 nm.

## 3. Results

### 3.1. Fast and Scalable Payload Removal Using Strong Cation Exchange Chromatography Membrane Adsorbers

**Figure 1:**
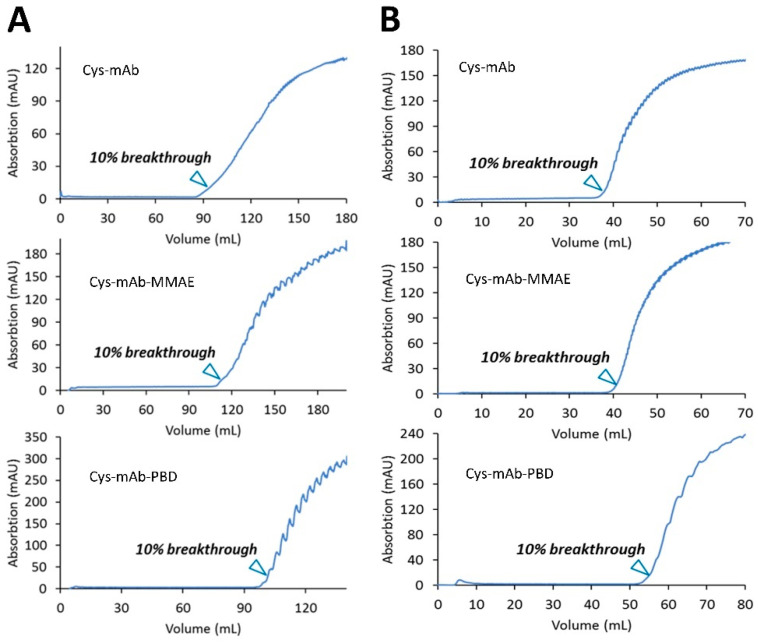
Membrane dynamic binding capacity at 10% breakthrough. (**A**) Sartobind^®^ S, 3 mL, 8 mm bed height with mAb, mAb-MMAE, and mAb-PBD conjugates. (**B**) Sartobind^®^ Phenyl, 3 mL, with mAb, mAb-MMAE, and mAb-PBD conjugates. Protein samples were diluted as 1.0 mg/mL with 20 mM MES, pH 6.0.

CEX membrane adsorbers (Sartobind^®^ S, 3 mL) were tested for the ability to remove free linker-payloads from the ADC product. First, the dynamic binding capacity (DBC) at 10% breakthrough was determined for three different loads: engineered cys-mAb (control) and cys-mAb conjugated to MMAE or PBD linker-payloads (synthesis described in the Materials and Methods section). The cys-mAb, MMAE, or PBD conjugates in reaction buffer (50 mM sodium phosphate, pH 7.0) at 3.5 mg/mL were diluted to 1.0 mg/mL with 20 mM MES pH 6.0 to adjust pH to 6.5 and loaded onto the membranes equilibrated 10 MVs of CEX equilibrium buffer (20 mM MES buffer, pH 6.0) at 1 MV/min. Breakthrough curves for each load (Figure 1A) were used calculate the DBC values summarized in Table 1. DBC values ranging from 32–37 mg/mL membrane volume (mg/mL) were measured, suggesting conjugation of PBD or MMAE linker-payloads to the cys-mAb results in only a minor variation of the protein’s charge profile.

**Figure 2:**
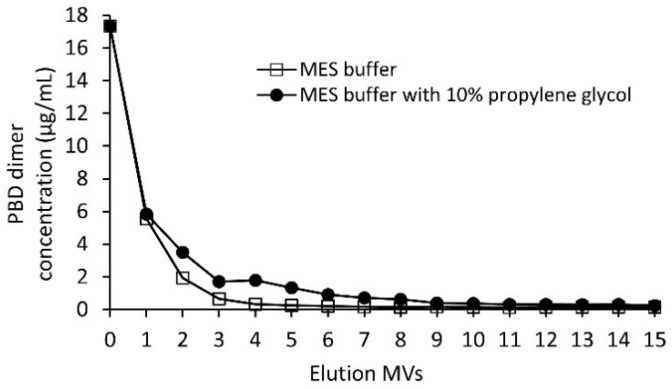
The Sartobind^®^ S clearance trend of the linker-PBD using 20 mM MES buffer at pH 6.0 with and without 10% propylene glycol. The linker-PBD concentration from each eluted MVs was quantified with the LC-MS/MS method as described in the Materials and Methods section.

Depending on the ADC conjugation process, the amount of free linker-PBD dimer present in the crude conjugation reaction mixture is usually in the range of 10–60 μg/mL. To evaluate the Sartobind^®^ S free drug clearance efficiency, 52 μg/mL linker-PBD in 50 mM sodium phosphate buffer (pH 7.0) was diluted with 20 mM MES buffer (pH 6.0) at a ratio of 1:2 to mimic the ADC sample loading conditions. This linker-PBD spiked solution was loaded onto a 3 mL, 8 mm Sartobind^®^ S membrane and washed with up to 15 MVs with either 20 mM MES buffer (pH 6.0) or the MES buffer with 10% propylene glycol. Samples were collected at each MV of the wash and analyzed for free drug. As shown in Figure 2, linker-PBD was detected at high levels early in the wash. After washing the membrane with 10 MVs, the amount of linker-PBD from eluted fractions dropped to less than 250 ng/mL with or without 10% propylene glycol, which is about 70 times reduction compared to the load.

**Figure 3:**
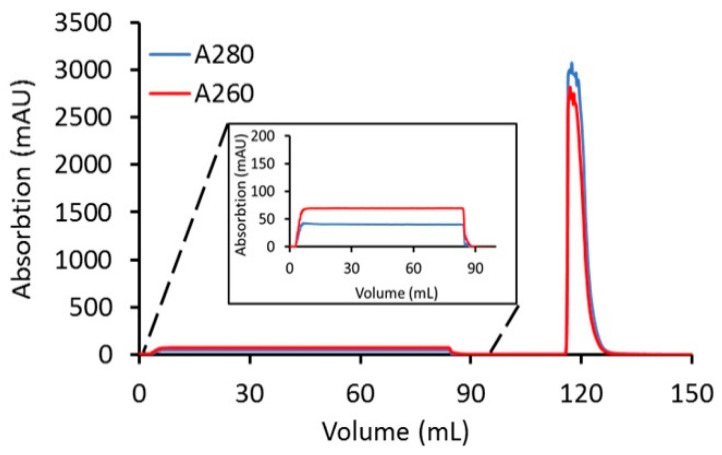
Clearance of linker-PBD from crude conjugation reaction mixture using the Sartobind^®^ S membrane. A total of 82 mg of cys-mAb-PBD crude reaction mixture (1 mg/mL) was loaded onto a 3 mL, 8 mm bed height Sartobind^®^ S membrane device at a flowrate of 1 MV/min. After loading the sample, the membrane was washed with 20 mM MES buffer at pH 6.0 for 10 MVs, and then the cys-mAb-PBD conjugate was eluted with 20 mM MES, 350 mM NaCl buffer, pH 6.0. The red line indicates the absorption at 260 nm; the blue line indicates the absorption at 280 nm.

**Figure 4:**
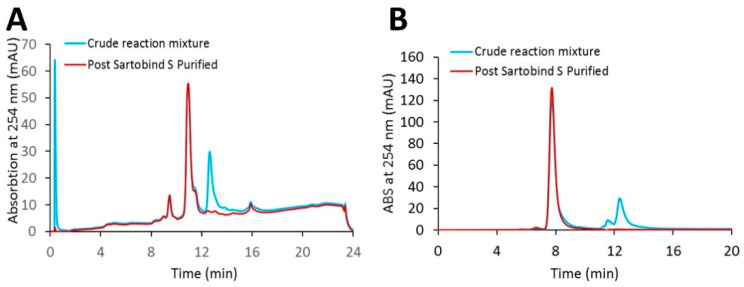
HPLC analytical characterization of the cys-mAb-PBD crude reaction mixture and the Sartobind^®^ S membrane purified conjugate. (**A**) HIC analysis. (**B**) SEC analysis. The blue line indicates the crude reaction mixture; the red line indicates the purified conjugate.

Using a loading ratio of 80% of the DBC, a bind-and-elute CEX purification run was used to test the removal of excess linker-PBD from a crude reaction mixture (Figure 3). A total of 82 mg of cys-mAb-PBD crude reaction mixture was loaded onto a 3 mL, 8 mm bed height Sartobind^®^ S membrane device at a flowrate of 1 MV/min. During the sample loading phase, a flow-through peak was observed with a higher absorption signal at 260 nm (A260) than 280 nm (A280), characteristic for small-molecule payloads, suggesting the free payload molecules (and other small-molecule impurities such as reaction cosolvent) do not bind the membrane during sample loading. The membrane was washed with 10 MVs of equilibrium buffer, prior to an isocratic elution phase with CEX elution Buffer (20 mM MES, 350 mM NaCl, pH 6.0). The eluate fraction was tested for protein content via UV–VIS, with a total of 82 mg of protein recovered, suggesting that 100% recovery of the conjugates was achieved. The starting material (crude reaction mixture) and the CEX-purified conjugates were analyzed for free linker-PBD using HIC, SEC, and LC-MS/MS methods. The small molecule peak, corresponding to free linker-PBD and cosolvent, in the analytical HIC and SEC chromatographs (Figure 4) was no longer observed in the CEX-purified material, suggesting small-molecule impurities were removed via CEX purification. Further quantification of free linker-PBD with the LC-MS/MS method suggested the amount of free linker-PBD was 78.42 ng/mL in the final eluted ADC solution at 9.1 mg/mL. The amount of free linker-PBD was about 0.046% on a molar basis relative to ADC.

**Figure 5:**
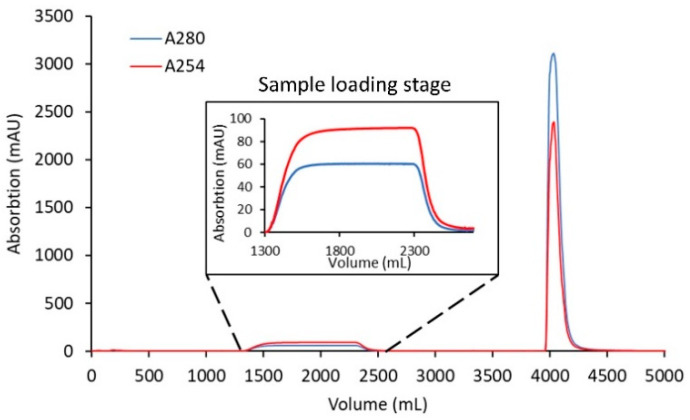
Scale-up clearance of linker-PBD from crude conjugation reaction mixture using the Sartobind^®^ S membrane. A total of 1.7 g of cys-mAb-PBD crude reaction mixture was loaded onto a 75 mL, 8 mm bed height Sartobind^®^ S membrane device at a flowrate of 1 MV/min. After loading the sample, the membrane was washed with 20 mM MES buffer at pH 6.0, and then the cys-mAb-PBD conjugate was eluted with 20 mM MES, 350 mM NaCl buffer, pH 6.0. The red line indicates the absorption at 254 nm; the blue line indicates the absorption at 280 nm.

To test the scalability of the CEX purification using membrane adsorbers to remove free payload from the conjugation crude reaction mixture at gram-scale, 1.7 g of cys-mAb-PBD in reaction buffer was diluted with CEX equilibration buffer (20 mM MES buffer, pH 6.0) to a concentration of 1 g/L and then loaded onto a 75 mL Sartobind^®^ S CEX membrane adsorber equilibrated with CEX binding buffer (20 mM MES buffer, pH 6.0). The membrane was washed with 10 MVs of CEX equilibration buffer, and conjugates were then eluted from the membrane with CEX elution buffer at a flow rate of 1 mV/min. Figure 5 shows the UV chromatogram from the scale-up run. The purified conjugate (eluate fraction) was analyzed by HIC, SEC, and LC-MS/MS. The HPLC chromatograms show that the payload was efficiently removed, and the process was scalable at 1.7 g of protein with the process being completed within 30 min. Residual free payload was measured using an LC-MS/MS assay (described in the Materials and Methods section). The residual payload amount was reduced from 88.8 µg/mL in the crude reaction mixture, to 63.81 ng/mL after washing with 10 MVs of CEX binding buffer, which is over 3 logs reduction of free linker-PBD.

### 3.2. Removal of Protein-Based Impurities Using Hydrophobic Interaction Chromatography Membrane Adsorbers

Membrane adsorbers (Sartobind^®^ Phenyl, 3 mL) with a Phenyl ligand for preparative HIC purifications were used to refine DAR distribution and remove high molecular weight species (HMWS) such as protein aggregates. The DBC at 10% breakthrough of Phenyl membrane adsorbers was determined for three different loads: engineered cys-mAb (control) and cys-mAb conjugated to MMAE or PBD linker-payloads (synthesis described in the Materials and Methods section). The cys-mAb or conjugates in 50 mM sodium phosphate at 3.5 mg/mL were diluted 4-fold with HIC binding buffer (25 mM sodium phosphate, 1 M ammonium sulfate, pH 7.0) to adjust the salt concentration prior to loading onto the membranes equilibrated with 10 MVs of HIC binding buffer at 1 MV/min. Breakthrough curves for each load (Figure 1B) were used to calculate the DBC values summarized in Table 1. DBC values ranged from 13–15 mg/mL membrane volume, with PBD conjugate, the most hydrophobic species of the loads tested, displaying a higher DBC.

**Figure 6:**
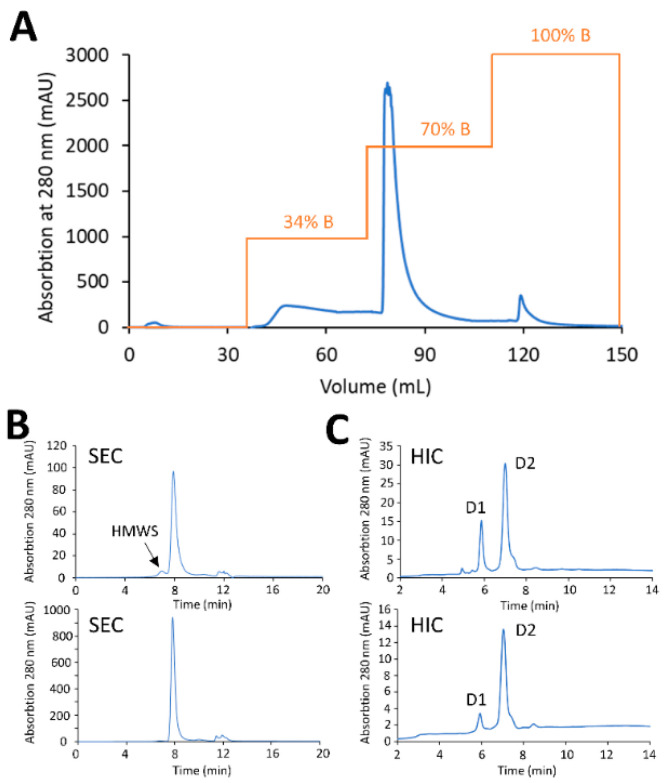
HIC purification of mAb-PBD ADC with the Sartobind^®^ Phenyl membrane. (**A**) Preparative purification with Sartobind^®^ Phenyl 3 mL, 8 mm bed height. The loaded sample was stepwise eluted with 34%, 70%, and 100% B. The desired DAR 2 specie was eluted with 70% B. (**B**) SEC profile showing the percentage of aggregate dropped from 5% to less than 1% after Sartobind^®^ Phenyl purification. (**C**) HIC profile showing the crude reaction mixture contains ADC variants with a range of DAR. The DAR of the purified ADC increased from 1.68 to 1.94.

Small-scale screening experiments were used to test the ability to remove protein-based impurities using preparative HIC membrane adsorbers. To determine the resolving power of phenyl membrane adsorber to remove under-conjugated species, a crude reaction mixture of PBD conjugates was generated to purposely have a high percentage of low-DAR species (antibody with zero drug, D0 and antibody with one drug, D1) representing a worst-case scenario. A total of 20 mg of crude PBD conjugate with a DAR of 1.68 and 4.9% HMWS (see the Materials and Methods section) were diluted 4-fold in HIC binding buffer and loaded at 1 MV/min onto a Sartobind^®^ Phenyl membrane (3 mL) equilibrated with HIC binding buffer. After sample loading, the membrane was washed with 3 MVs of equilibration buffer, prior a multi-step elution phase into HIC elution buffer (25 mM sodium phosphate, pH 7.0, with 20% (*v/v*) isopropyl alcohol (IPA)). Elution of the low-DAR species was carried out using an initial step-gradient to 34% elution buffer for 10 MVs, after which a second step-gradient to 70% elution buffer for 10 MVs was used to elute the target DAR 2 conjugate, and finally a step -gradient to 100% elution buffer for 10 MVs was used to strip off HMWS impurities, including higher-DAR and aggregates (Figure 6A). Analysis by analytical HIC of the purified conjugates from the target fraction (eluates at 70% elution buffer) displayed a final DAR of 1.92, denoting significant purification of the desired DAR 2 species. In addition, the HMWS decreased from 4.9% to less than 1% when analyzed by SEC (Figure 6B,C).

**Figure 7:**
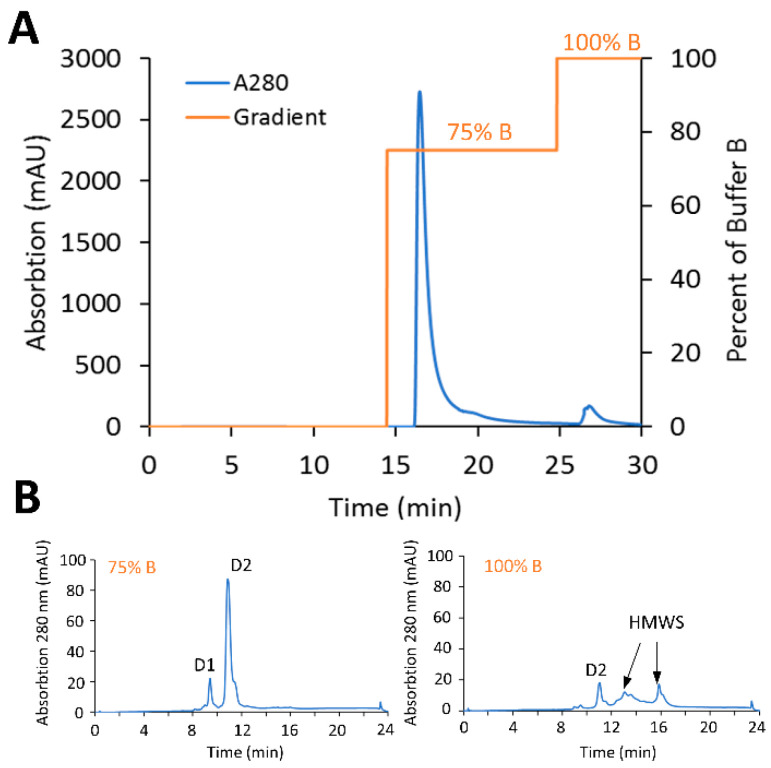
Scale-up purification with the Sartobind^®^ Phenyl membrane. (**A**) Purification of engineered Cysteine-mAb-PBD ADC using Sartobind^®^ Phenyl in the stand-alone model. The Sartobind^®^ S purified material (gram-scale) was loaded to a 150 mL Sartobind^®^ Phenyl membrane then targeted, and undesired ADC species were eluted sequentially. (**B**) HIC profile of 75% B purified ADC. The HMWS was flushed out with 100% B.

The scalability of the preparative HIC purification using the Phenyl membrane to refine DAR distribution, as well as removal of HMWS, was then assessed at gram-scale with PBD-based conjugates. The gram-scale CEX-purified material (1.7 g, DAR 1.82, 5% HMWS) described above was diluted 4-fold with HIC binding buffer and loaded onto a Phenyl membrane (150 mL, 8 mm bed height, ca. 75% DBC of phenyl membrane) equilibrated with HIC binding buffer at 1 mV/min. Given the lack of low-DAR species in the crude HIC load material, after a 10 MVs wash with HIC binding buffer, a step-gradient to 75% elution buffer 5 MVs in length was used to elute target species, and a final step-gradient at 100% elution buffer was used to strip the HMWS (Figure 7A). The target elution fraction contained 1.45 g (85% total yield) of final purified ADC was recovered with a DAR of 1.82 determined by HIC and less than 1% by SEC-HPLC (Figure 7B). These results demonstrate the ability of the Sartobind^®^ membrane adsorbers to remove protein-based impurities at gram-scale and that operation could be completed within 30 min, while maintaining a high yield.

### 3.3. Tandem Model Chromatography for Removal of Small and Large Molecule Impurities

CEX and HIC chromatography are complimentary methods for multi-dimensional chromatography [16,17,18]. The ability to save processing time and simplify production by removing small molecule-based (free payload, cosolvent, quencher) and large molecule-based (non-target DARs, aggregates) impurities in a single unit operation was investigated.

**Figure 8:**
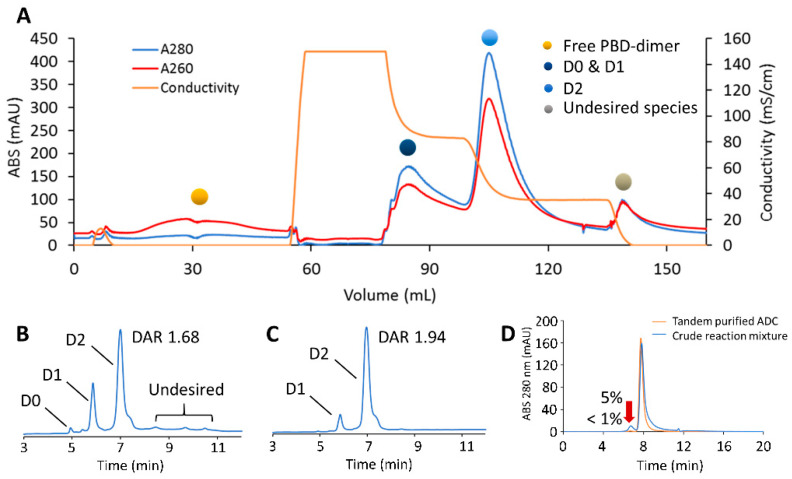
Tandem-model purification of the crude mAb-PBD dimer conjugate. (**A**) Purification of engineered Cysteine-mAb-PBD ADC in the tandem model. The quenched reaction mixture was loaded to Sartobind^®^ S that was tandemly connected to Sartobind^®^ Phenyl. The conjugation species were separated through loading, washing, and multiple elution steps. (**B**) HIC profile showing that the crude reaction mixture contains ADC variants with a range of DAR, residual linker-PBD, aggregates, and organic solvent. (**C**) HIC profile of the tandem method-purified ADC. The DAR of the ADC increased from 1.68 to 1.94. (**D**) SEC profile showing the percentage of aggregate dropped from 5% to less than 1% after tandem-model purification.

For these experiments, a CEX (Sartobind^®^ S, 3 mL, 8 mm bed height) membrane followed by a HIC (Sartobind^®^ Phenyl, 3 mL, 8 mm bed height) membrane were placed in tandem. The membrane adsorbers, which are manufactured as self-enclosed capsules, were sequentially connected using a male-to-male luer adaptor. A total load of 30 mg of mAb-PBD ADC (crude DAR 1.68 with 5% HMWS; see the Materials and Methods section) were diluted 3-fold in CEX binding buffer (20 mM MES, pH 6) and loaded at 1 MV/min onto the tandem assembly membrane, which was equilibrated with CEX binding buffer. During the loading stage, all conjugate species were captured by the CEX membrane, while small-molecule impurities flowed through the membranes. After loading was complete, the conjugate species bound to the CEX membrane were then washed with CEX binding buffer followed by elution using HIC binding buffer for 5 MVs. During this phase, the chromatograph (Figure 8A) indicated that no protein-related species were eluted from the tandem membranes as the conjugate species desorbed from the CEX membrane were sequentially captured by the HIC membrane. Target DAR 2 conjugates were then purified from large-molecule impurities through a series of three isocratic step-gradients with 34%, 70%, and 100% HIC elution buffer. Fractions that eluted with 70% HIC elution buffer step-gradient were collected as the purified ADC fraction and analyzed.

Analytics of the tandem purified fraction showed a final DAR of 1.94 was obtained, with less than 1% HMWS by SEC (Figure 8B–D). With a total processing time of 45 min and a yield of 80% DAR 2 species, this tandem multi-dimensional chromatography approach provides an innovative one-step solution for the purification of complex ADC mixtures using membrane adsorbers.

## 4. Discussion

Since the toxic payloads used in ADC processes are highly potent, the level of acceptable free payloads present in the purified ADCs usually range from 1 to 0.1% on a molar basis relative to the ADCs [6,15]. These purity levels are difficult to achieve via a simple UF/DF purification, and often require extended diavolumes with additives or activated carbon filter to meet residual payload specifications [5]. Bind and elute chromatography is usually an applicable method to remove free payloads from ADCs. Here, a fast and scalable CEX approach is used to purify excess linker-PBD from a crude reaction mixture.

To evaluate the clearance of the free linker-PBD, the sample spiked with linker-PBD at 52 µg/mL was subjected to a Sartobind S membrane purification as described above. The linker-PBD was detected at high levels in initial eluted fractions, and the amount of linker-PBD rapidly fell after 10 MVs of washing. In general, propylene glycol is commonly used in ADC production to increase the solubility of hydrophobic payload [19,20]. The washing process seem to be highly efficient with or without propylene glycol, as the free linker-PBD concentrations reached less than 250 ng/mL after 10 MVs with both washing conditions. Considering the potential risk of lost materials using propylene glycol in the washing stage, the following study was carried without propylene glycol. With the linker-PBD clearance study carried out at 3 mL and 75 mL membrane scales, starting at around 88.8 µg/mL in the crude conjugate solution, the free payload was cleared efficiently with final concentration at 78.42 ng/mL and 63.81 ng/mL, respectively. In both cases, after the Sartobind S membrane purification, the amount of free payload was less than 0.1% on a molar basis relative to ADC. It is worth mentioning that the amount of free linker-PBD observed here was lower than the initial clearance study, possibly due to the conjugates competing with free linker-PBD on binding to the CEX membrane that impacted differently for the collected. The purification of crude conjugates at gram-scale was achieved within 30 min using a strong cation exchange ligand. The measured DBCs were similar to conventional resin media with sulphonate ligands, although the total processing time was drastically shortened, which translates to lower manufacturing costs. Overall, our experiments demonstrated that the Sartobind^®^ S membrane could efficiently and reliably remove the free linker-PBD from the crude ADC reaction mixture. The residual linker-PBD content level was below the standard manufacturing requirement as well.

Given the highly hydrophobic nature of payload molecules commonly used for ADC indications (e.g., PBD dimers, MMAE, etc.), polishing of protein-based impurities like non-target DAR species often employ preparative HIC chromatography resin media for purifications [9]. Phenyl Sepharose High Performance (GE Healthcare) serves as a widely used stationary phase in the field given its small bead size (~34 µm diameter) and high ligand density. To evaluate the performance of membrane absorber to purify ADCs, the Sartobind^®^ Phenyl membrane has been successfully utilized to purify mAb-PBD dimer DAR 2 conjugates in a bind/elute mode from mg to gram-scale. The Phenyl membrane has a dynamic binding capacity comparable to currently available HIC resins used in many ADC processes, and shows excellent resolution in isolating the target DAR species from the under-conjugated species and HMWS.

In the present work, the classic preparative HIC purification conditions were adapted to membrane chromatography to polish the crude mAb-PBD dimer conjugates with over 80% yield, and obtained a final HMWS level below 1% for the purified ADC samples. HIC purification was first carried out in a linear gradient to evaluate the performance of membrane separation. Since a good resolution was achieved with a linear-gradient method, in order to develop a fast and simple process for ADC polishing, an isocratic elution strategy was implemented in this study. We found that isocratic elution gave a shorter overall process time, with similar results for purified ADC compared to gradient elution, but without sacrificing the ADC yield and purity. Based on these results and related work, we have shown that impurities and target large molecules can be efficiently eluted off the membranes with 5–10 MVs. We conclude that the isocratic elution resulted in overall reduction in the processing time, and reduction in the overall amount of buffer used in the process. However, we believe the purification method can be further optimized by using several mobile phase modifiers [21]. Mobile phase modifiers can promote desorption or promote solubility of bound species from the membrane ligand, while maintaining all parameters constant (loading ratio, flow rate, etc.) and changing only the additive used in the elution buffer to either IPA, glycerol, propylene glycol, or dimethylacetamide. The use of glycerol or propylene glycol in the elution buffer could be a feasible alternative to IPA and improve manufacturability, as some facilities may be incompatible with organic solvents.

Finally, an innovative tandem-membrane purification approach was demonstrated by connecting the Sartobind^®^ S and Phenyl membranes in series. Although two-dimensional liquid chromatography has been developed at the analytical chemistry level to address difficult-to-resolve mixtures [17,18], few applications have been reported to address large-molecule purification challenges. One of the hurdles inherent in resin-based chromatography is the high backpressure caused by connecting two different separation columns in sequence. The mixed-mode resin could be an alternative option for resin-based chromatography; however, developing a purification method using mixed-mode resin is not straight forward, not to mention the resin packing and cleaning may be problematic as well. Instead, the tandem-membrane purification provides a new insight to address those issues and fills in the gaps left by resin-based conventional ADC process.

The main advantage of membrane chromatography for purification of large biomolecules is attributed to convective-driven transport; affording the potential to operate at high flow rates and low pressure [10]. Since the CEX elution and HIC binding conditions are complimentary, the two separate membrane-purification steps can be combined by connecting membranes in sequence without redeveloping the purification method. Using the same bind/elute conditions developed in CEX and HIC membrane chromatography, we were able to achieve similar results (free linker-payload and HMWS removal, DAR, yield) with tandem-membrane chromatography.

## 5. Conclusions

**Figure 9:**
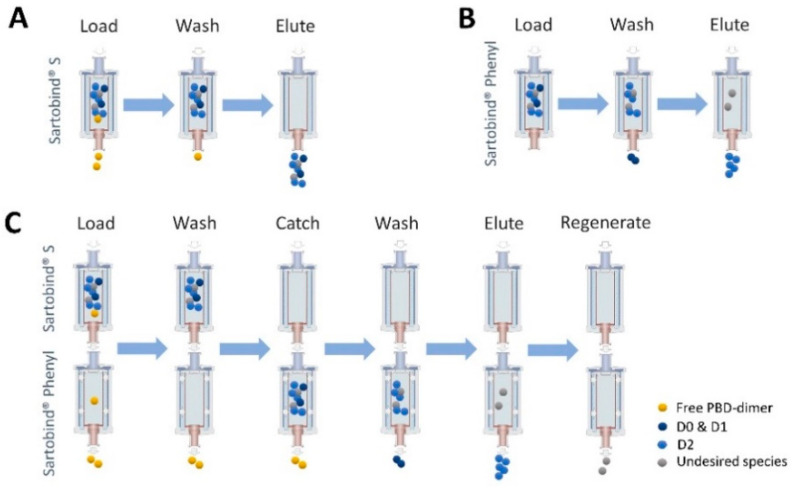
Process-diagram overview. (**A**) Bind/Elute using Sartobind^®^ S to remove residual payload. (**B**) Bind/Elute using Sartobind^®^ Phenyl to remove aggregate in stand-alone model. (**C**) Simplified process to purify target ADC with Sartobind S and Phenyl in tandem model.

Antibody–drug conjugates are a fast-developing field of cancer therapeutics, or theranostics. ADC purification process development is complicated by the growing diversity in the drugs, conjugation chemistries, and molecule designs used for ADC production. Comparing to the conventional resin-based chromatography and UF/DF purification strategies, a new type of membrane-based purification processes was developed and summarized in Figure 9. The Sartobind^®^ S and Phenyl membrane adsorber presents a new opportunity in ADC processing. The membrane-based process consumes less buffer and shortens processing times, which reduces the cost and time of ADC bioprocess and cGMP manufacturing campaigns. Membrane devices are scalable, single-use, closed systems that improve manufacturing safety, and eliminate the need for packing, qualification, and cleaning validation studies associated with resin-based column chromatography. Removal of free payload, aggregates, and refinement of drug distribution profile of an ADC by membrane chromatography presents a novel and efficient process that directly translates into improved efficiency during both process development and cGMP manufacturing. Sartobind^®^ S efficiently removes the free payload from crude ADC reaction mixture. Sartobind^®^ Phenyl was used successfully to refine DAR and to remove HMWS including aggregates. Sartobind^®^ S and Phenyl in tandem efficiently removes free payload, refines DAR and removes aggregate in a single-unit operation. Membrane purification could be a platform ADC manufacturing process to be used as a starting point for ADC development.

## Figures and Tables

**Table 1:** Membrane dynamic binding capacity.

	Sartobind^®^ S	Sartobind^®^ Phenyl
Load	DBC, 10% Breakthrough	DBC, 10% Breakthrough
mAb	32 mg/mL	13 mg/mL
mAb-MMAE	37 mg/mL	14 mg/mL
mAb-PBD	34 mg/mL	14.9 mg/mL

## Data Availability

Not applicable.
